# Numerical study on the start and unstart phenomena in a scramjet inlet-isolator model

**DOI:** 10.1371/journal.pone.0224994

**Published:** 2019-11-07

**Authors:** Jaewon Lee, Sang Hun Kang

**Affiliations:** 1 Department of Mechanical Engineering, Sejong University, Seoul, Korea; 2 Department of Aerospace System Engineering, Sejong University, Seoul, Korea; Coastal Carolina University, UNITED STATES

## Abstract

Inlet unstart and buzz in scramjet engines must be prevented for the stable operation of the engines. In the present study, the characteristics of the inlet start, unstart and buzz phenomena in a scramjet engine inlet model are investigated using numerical analysis with the RANS-based OpenFOAM solver. The results for the inlet start case with a small computational domain that includes only the inlet-isolator part are in good agreement with existing numerical and experimental results. However, for the inlet unstart case, the computational domain must be wide enough to consider the interactions between the upstream of the inlet and the internal flow of the inlet to predict the inlet unstart and buzz phenomena in the inlet test model. The present results show fairly good agreement with existing experimental results with the buzz phenomenon. The effects of boundary layer profiles on the buzz oscillation frequency and amplitude are also addressed.

## Introduction

By removing the compressor and turbine from gas turbine engines, scramjet engines obtain advantages such as compact structures and high-speed capabilities. However, due to the absence of the compressor, which adjusts the incoming air to be appropriate for reaction in the combustor, difficulties in controlling the incoming flow to the combustor can be weaknesses of scramjet engines [1, 2]. In particular, if the boundary layer grows within the inlet or the combustion pressure in the combustor increases, the shock wave structures within the inlet may become unstable, and the direction of the incoming air can be reversed. Depending on the cases, these phenomena may eventually result in inlet unstart and buzz problems. The inlet unstart problem is well known to be one of the main causes of various scramjet engine flight test failures [3–6]. Therefore, prevention of inlet unstart is an essential task for the successful development of ramjet and scramjet engines.

Inlet unstart problems have long been studied due to their importance and complexities [7–23]. The characteristics of the unstart phenomenon are slightly different from those of the hypersonic inlet of a scramjet engine and the supersonic inlet of a ramjet engine. In the supersonic inlet, normal shock occurs in the isolator, and subsonic combustion occurs in the combustor. Therefore, the pressure oscillations in the combustor are properly blocked by the normal shock waves, which give a relatively robust characteristic against the unstart phenomenon [7, 8]. However, in the hypersonic inlet of a scramjet engine that generates supersonic combustion in the combustor, the pressure oscillations in the combustor should be blocked by only the oblique shock waves and the shock trains [7, 8]. Therefore, it can be more difficult to control unstart problems in a hypersonic inlet than in a supersonic inlet. Furthermore, the airflow captured by an unstarted hypersonic inlet is partially and temporarily supersonic. Due to the temporary existence of the supersonic zone in the hypersonic inlet, the acoustic waves should be blocked temporarily. Therefore, it is difficult to predict the buzz oscillation frequency and amplitude in the hypersonic inlet [7–11].

Many studies have been performed regarding the inlet unstart phenomena in supersonic inlets [15–18]. Among those studies, Trapier et al. performed a numerical analysis of the inlet unstart and buzz phenomena of a supersonic inlet using DDES (Delayed Detached Eddy Simulation) [16]. Their numerical results are in good agreement with the buzz oscillation frequency and amplitude in their own experimental results [16, 17]. Many studies have been performed on unstart problems for hypersonic inlet cases as well [19–24]. However, it is hard to find a numerical study that accurately predicts the oscillation frequency and amplitude in an unstarted hypersonic inlet [8–9, 21].

Wagner experimentally studied the flow characteristics of a model scramjet engine inlet in Mach 5 conditions using a blowdown wind tunnel [22]. To simulate the back pressure behind the engine isolator, a flap was installed at the end of the model. By changing the angle of the flap, they investigated the characteristics of the inlet unstart and buzz phenomena. In their results, depending on the test conditions, the inlet start, unstart and buzz phenomena were observed. Wagner’s test results were numerically studied by Koo and Raman as well [21]. Using LES (Large Eddy Simulation), Koo and Raman studied the flow characteristics of the inlet start and unstart cases observed in Wagner’s experiments. Their numerical results accurately predicted the steady flow patterns in the inlet start case. However, even with the conditions for the inlet unstart, the inlet buzz phenomenon, which was observed in Wagner’s experiments, did not appear in their numerical results.

Although LES is more accurate than DDES, DDES accurately predicted the buzz in a supersonic inlet, whereas LES was not sufficiently accurate to reproduce the buzz in a hypersonic inlet [16, 21]. This finding implies that there must be another reason for the discrepancy other than the accuracy of the numerical method. This reason may be related to the mechanisms of inlet unstart phenomena.

Therefore, in the present study, we perform a numerical analysis using a widely used RANS (Reynolds-averaged Navier-Stokes equations)-based model to investigate the flow characteristics of the inlet start and unstart phenomena observed in Wagner’s experiments. We also identify important factors to accurately predict inlet buzz phenomena in hypersonic inlets.

## Numerical method

The open-source CFD software OpenFOAM is used to model the inlet start, unstart and buzz phenomena in a scramjet inlet-isolator model. The density-based compressible flow solver rhoCentralFoam is employed to solve the Navier-Stokes equations, and the k-ω SST (shear stress transport) model is used to consider the turbulent effect [25, 26]. In the numerical analysis, the first order implicit Euler scheme is used to calculate the time derivatives, and the central-upwind KNP (Kurganov, Noelle and Petrova) scheme and the van Leer limiter are used to evaluate the flux at cell faces [27, 28]. Sutherland’s viscosity model [29] is used to consider the temperature effects on the viscosity because the growth of the boundary layer by viscous effects is an important factor in the inlet start and unstart phenomena. Additionally, thermal property data, such as thermal conductivity and heat capacity, are taken from the JANAF table [30].

**Fig 1:**
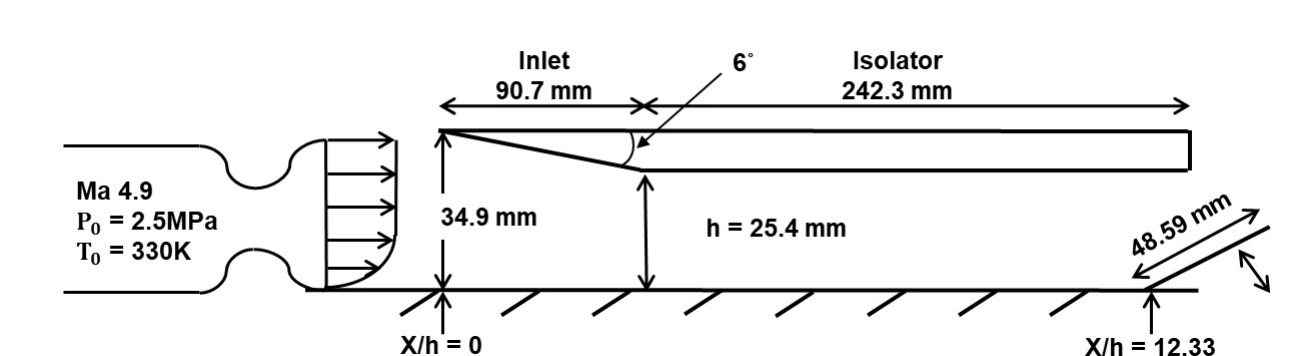
Schematic of the scramjet inlet isolator.

Fig 1 shows an illustration of the scramjet inlet-isolator test model [22]. As shown in the figure, the nozzle flow enters the inlet with a Mach number of 4.9, a total pressure of 2.5 MPa and a total temperature of 330 K. To simulate the back pressure behind the engine isolator, a flap is installed at the end of the model. Additionally, from the experimental data, a 19.05 mm-thick turbulent boundary layer is included in the incoming nozzle flow.

**Fig 2:**
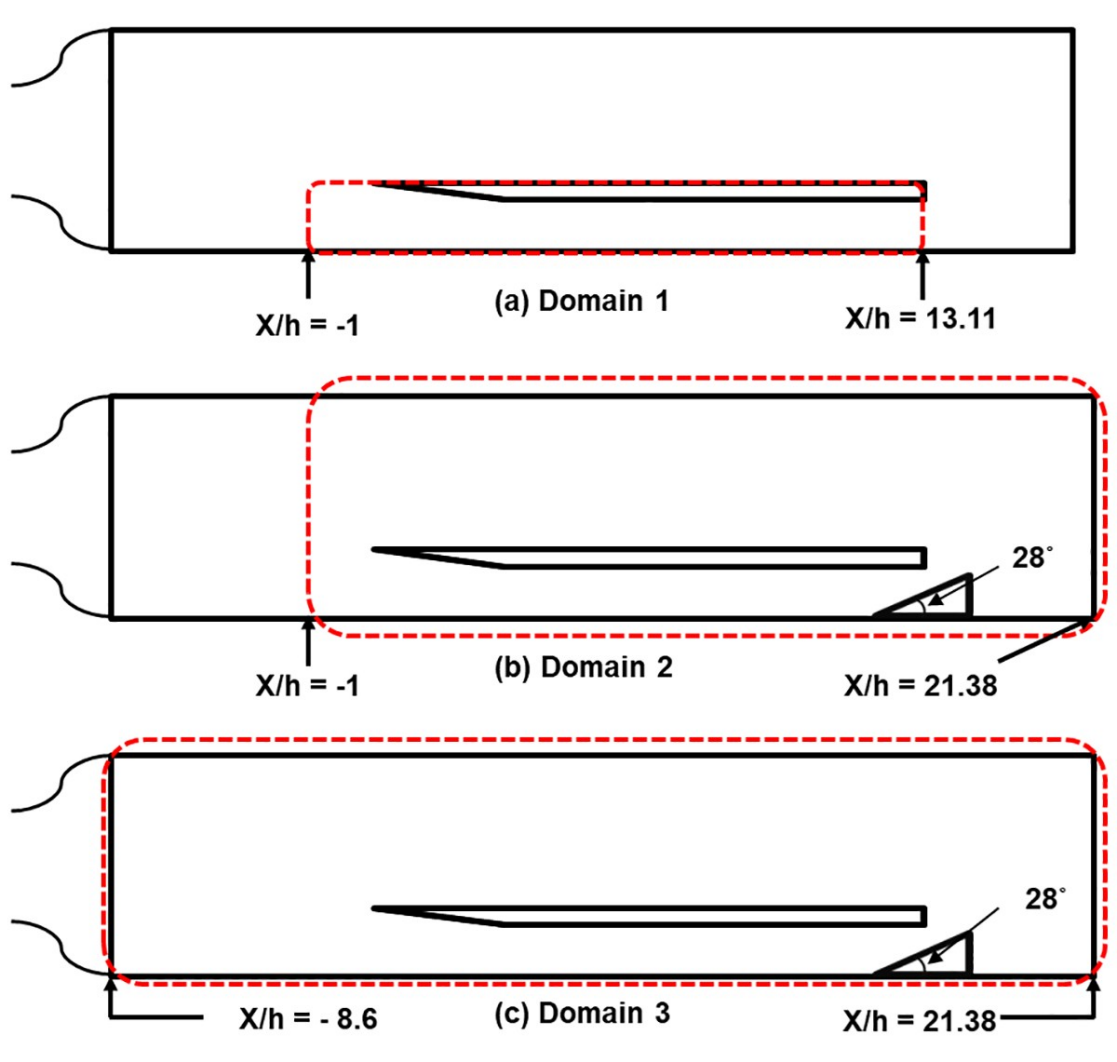
Configurations of the computational domain. (a) Domain 1. (b) Domain 2. (c) Domain 3.

Fig 2 shows the configurations of the computational domain for analyzing the flow characteristics of the test model. As shown in this figure, three different computational domains are used for the numerical analysis. Domains 1 and 2 are the same configurations as those used in the LES by Koo and Raman [21]. Domain 3 is an extended version of Domain 2 that includes the region near the Mach nozzle exit.

**Fig 3:**
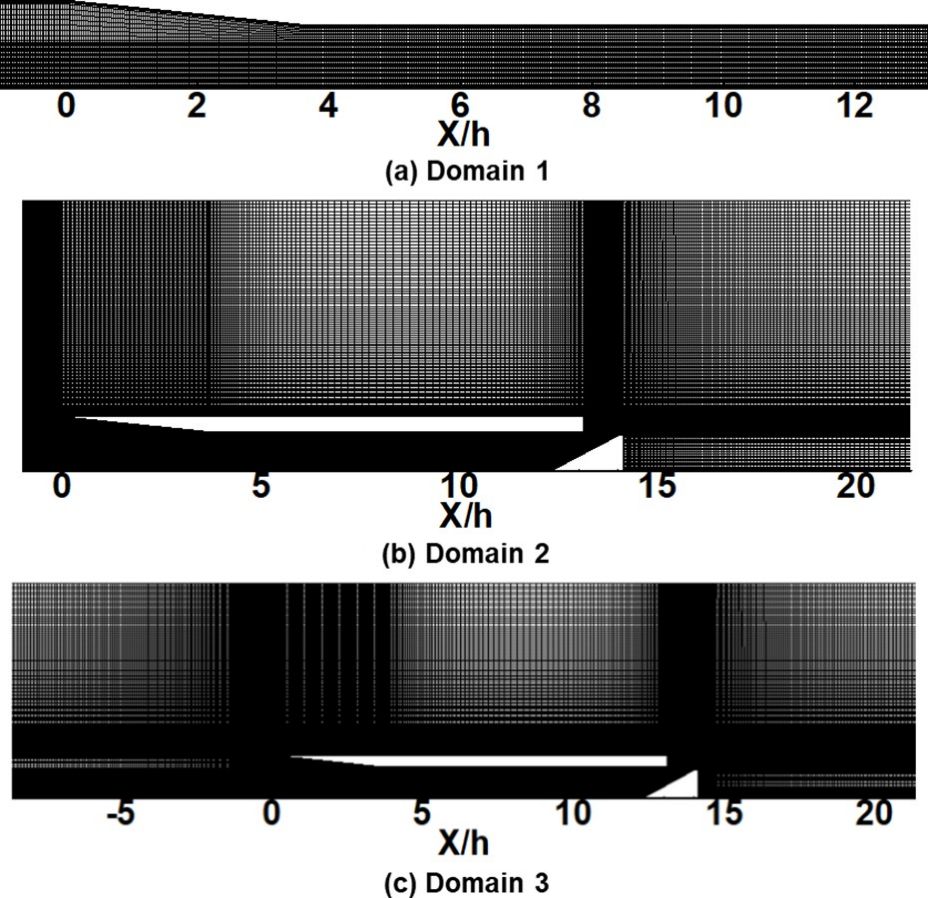
Grid system of the scramjet inlet isolator. (a) Domain 1. (b) Domain 2. (c) Domain 3.

Fig 3 shows the grids used to model the scramjet inlet isolator for each computational domain in Fig 2. Approximately 23,000 grid points are used in the mesh for Domain 1, approximately 99,000 for Domain 2, and approximately 81,000 for Domain 3. A grid resolution analysis showed that doubling the number of grid points did not significantly affect the results.

The common inflow boundary conditions based on the experimental data [22] are shown in Table 1. The supersonic outflow condition is used at the outlet of the domain. The velocity profiles for the 19.05 mm-thick boundary layer in the incoming nozzle flow are calculated by using turbulence boundary layer theory [31, 32]. All walls are assumed to be adiabatic.

**Table 1:** Inlet conditions of the scramjet inlet isolator [22].

P_t_ (MPa)	T_t_ (K)	M_0_	δ_99_ (mm)	Turbulent Intensity (%)
2.5	330	4.9	19.05	0.3

## Results and discussion

### Flow analysis of inlet start (Domain 1)

The flow characteristics for the inlet start case of the scramjet inlet-isolator model are investigated using the OpenFOAM program. As used in the LES study [21], Domain 1 (x/h = -1~13.11) is employed for the analysis of the inlet start. As in the experiment, the flap at the end of the model is completely folded to make the inlet start without high back pressure. Despite using the unsteady solver rhoCentralFoam, the flow characteristics of the present cases exhibit a steady state. Therefore, in the following figures, the obtained steady state data are presented after a long enough time.

**Fig 4:**
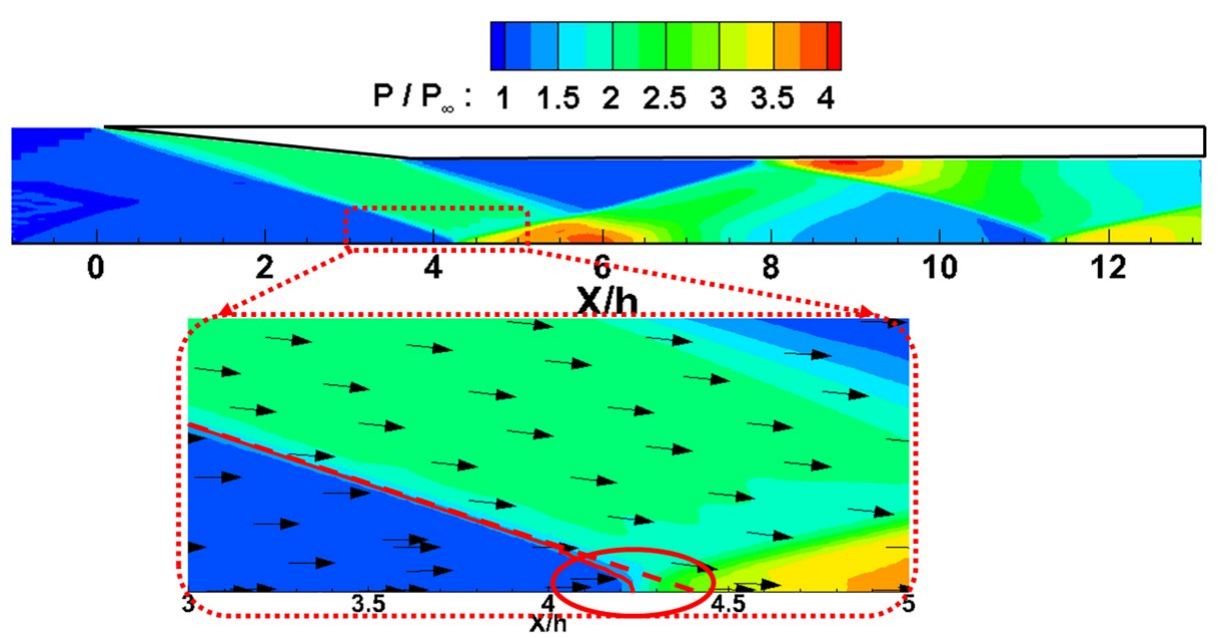
Normalized pressure contours of the inlet isolator for the start case.

Fig 4 shows the pressure distribution within the inlet isolator. As shown in this figure, the shock wave from the inlet cowl is reflected on the lower wall, and the reflected shock is reflected again on the upper wall. Additionally, an expansion wave is generated by the end of the upper cowl and reflected in the same way. At the point of the shock wave reflections on the lower wall (dotted area in the figure), it can be observed that the reflected shock wave angle is bended due to the boundary layer. However, since there is no obstacle behind the model to increase the back pressure and the oblique shock from the cowl is not so strong, no separation zone or recirculation zone is observed within the inlet, and the inlet maintains the starting state.

**Fig 5:**
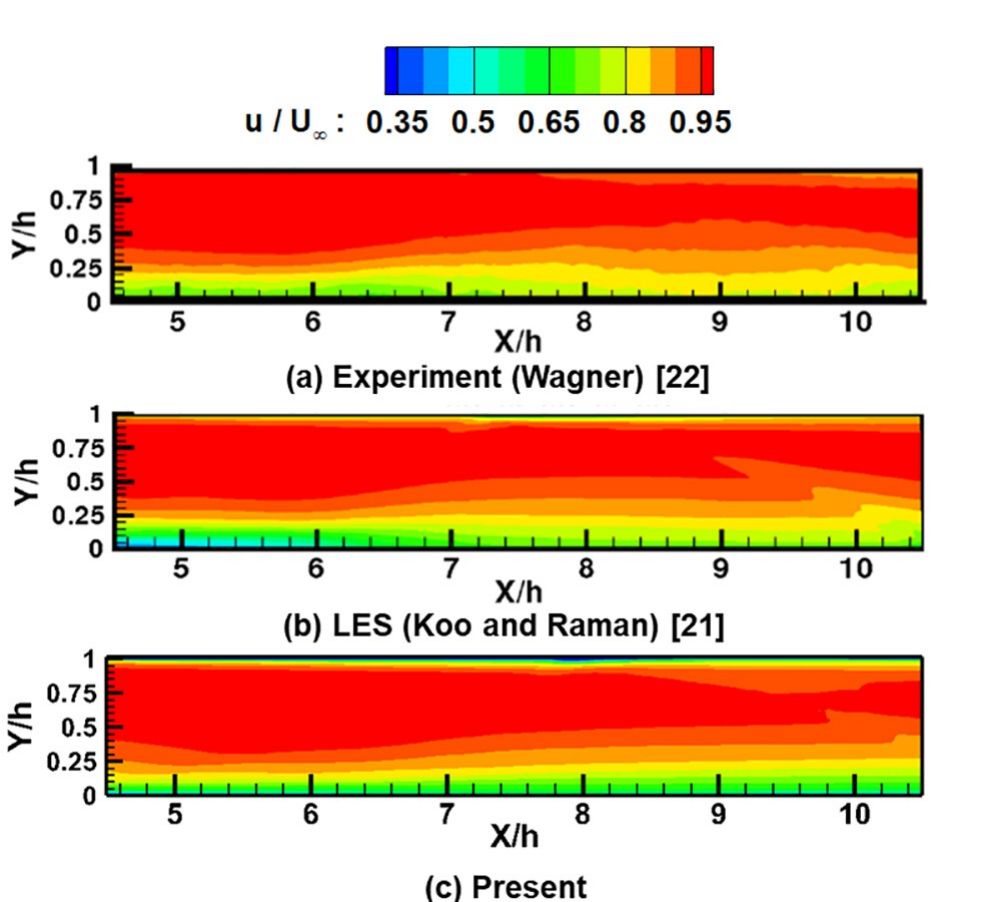
Normalized axial velocity contours of the inlet isolator for the start case. (a) Experiment (Wagner) [22]. (b) LES (Koo and Raman) [21]. (c) Present.

**Fig 6:**
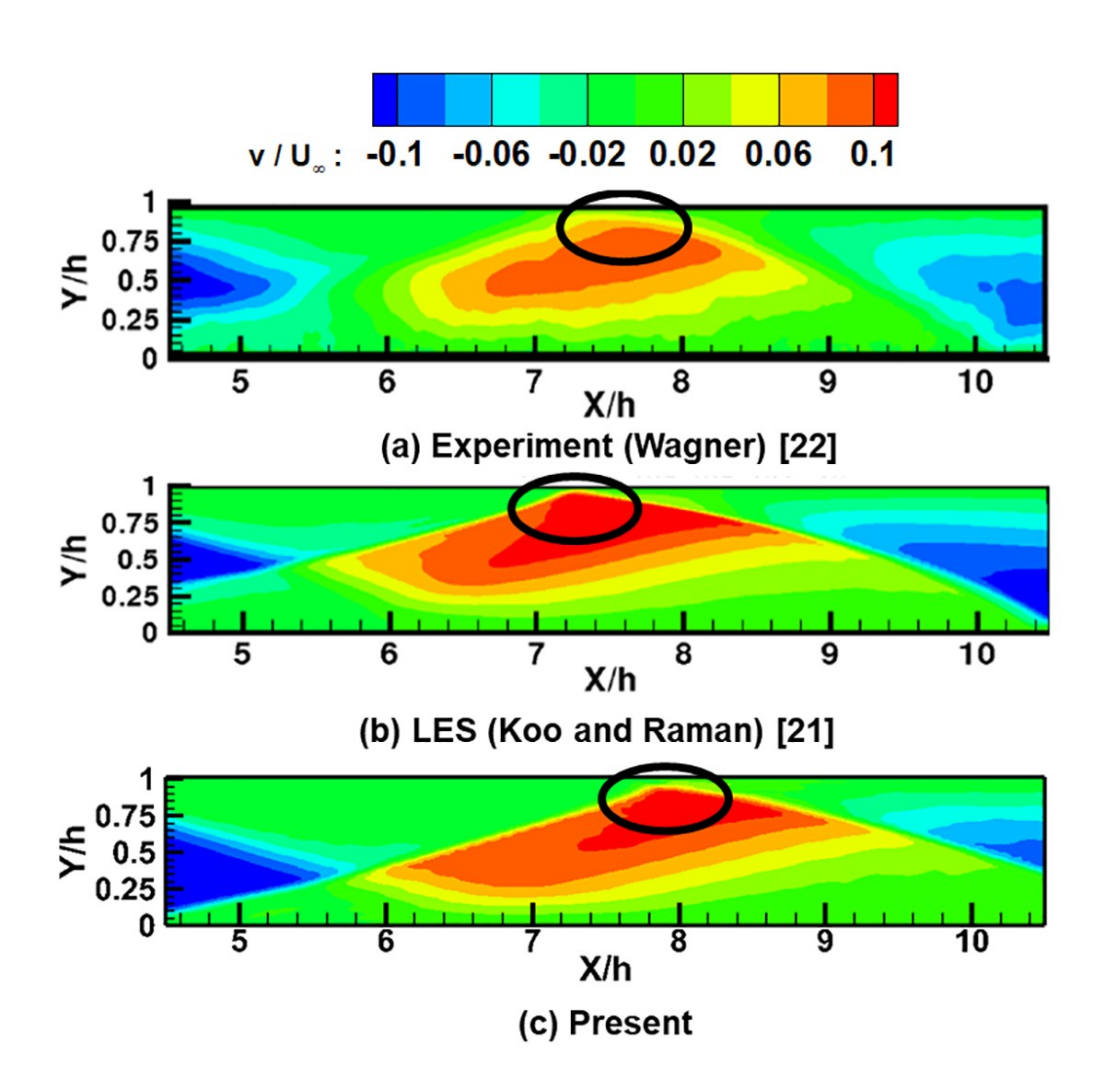
Normalized normal velocity contours of the inlet isolator for the start case. (a) Experiment (Wagner) [22]. (b) LES (Koo and Raman) [21]. (c) Present.

To confirm the accuracy of the present results, the normalized axial and normal velocity distributions are compared with experimental results [22] and LES results [21] in Fig 5 and Fig 6.

Fig 5 shows the distribution of the normalized axial velocity within the isolator part (x/h = 4.5~10.5). As shown in Fig 5, the results of this study are in good agreement with the results of existing experiments and LES. However, in the downstream section of the inlet, the present results show a somewhat thicker boundary layer than the experimental and LES results. According to previous studies, RANS-based numerical analyses using the k-ω SST model slightly overestimate the boundary layer thickness and the recirculation zone if shock-boundary interactions exist [33, 34]. Nevertheless, except in those specific regions, the present results are consistent with the experimental and LES results.

Fig 6 shows the distribution of the normalized normal velocity within the same area as shown in Fig 5. As shown in the figure, shock waves and expansion waves are reflected in the isolator, and due to those reflections, the flow repeatedly moves up and down within the isolator. The same patterns are also observed in the experimental and LES results. However, because of the numerical errors in the shock-boundary layer interaction explained above, the locations of the shock reflections on the upper wall (the circles in the figure) are different in the numerical results and experimental results.

**Fig 7:**
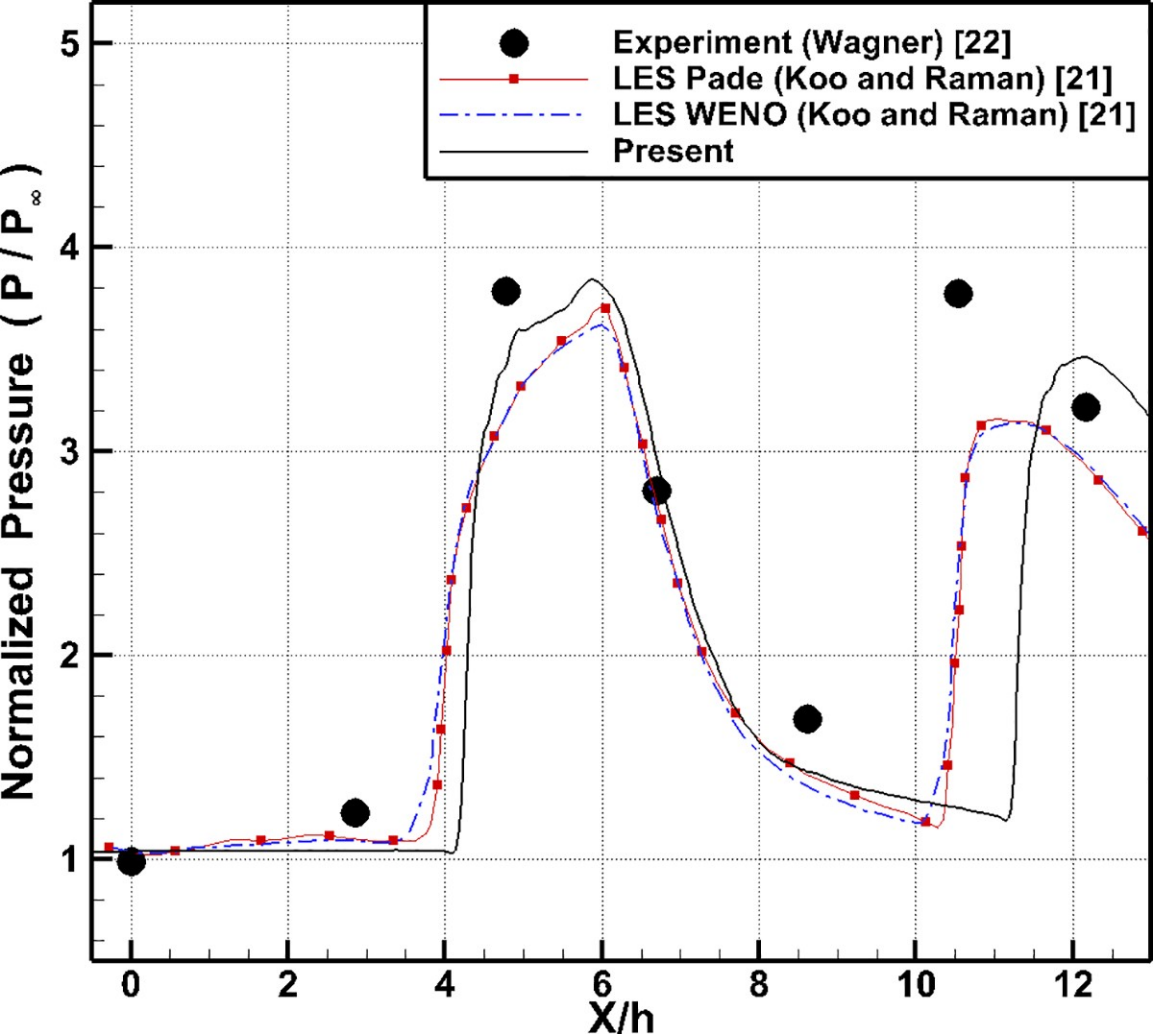
Normalized lower wall pressure distribution of the inlet isolator.

To verify the accuracy of the present results more quantitatively, the normalized lower wall pressure distribution of the inlet-isolator model is compared with that of the experimental and LES results in Fig 7. As shown in this figure, the first peak point of the lower wall pressure predicted by the present analysis is in good agreement with that of the experimental and LES results. However, the location of the second peak point predicted by the present analysis is approximately (1.7 x/h) behind the corresponding point in the experimental results. The location of the second peak point predicted by the LES results is similar to that in the experimental results; however, the magnitude of the second peak point predicted by the LES results is approximately 16% smaller than that in the experimental results. Thus, as previously described, it can be reconfirmed that the error in the shock-boundary layer interaction accumulates as it moves downstream.

From the above results, except for the typical error factors that can be observed in various numerical analyses including the LES, the present analysis accurately predicts the flow characteristics of the scramjet inlet-isolator model at steady state.

### Flow analysis of inlet unstart (Domain 2)

In the previous section, the present numerical methods were proven to be accurate enough for the cases with steady state start flow in the inlet-isolator model. In this section, using these confirmed numerical methods, the unsteady behaviors of the unstart flow in the inlet-isolator model are investigated. As in the LES study [21], Domain 2 (x/h = -1~21.33) is employed as the computational domain. The flap is installed at the end of the model to simulate combustion pressure and induce inlet unstart. In the experiments, the flap angle was slowly increased or decreased to observe changes in the flow characteristics. Depending on the flap angle, three types of unstart modes were observed in the experimental results. At the maximum flap angle, the high-amplitude oscillatory mode was observed. Upon reducing the flap angle, the high-amplitude oscillations were reduced to the non-oscillatory mode. Finally, a slight increase in the flap angle resulted in the lower-amplitude oscillatory mode. In the present study, we assume that the flap angle is fixed at the maximum value (28°) for compact analysis.

**Fig 8:**
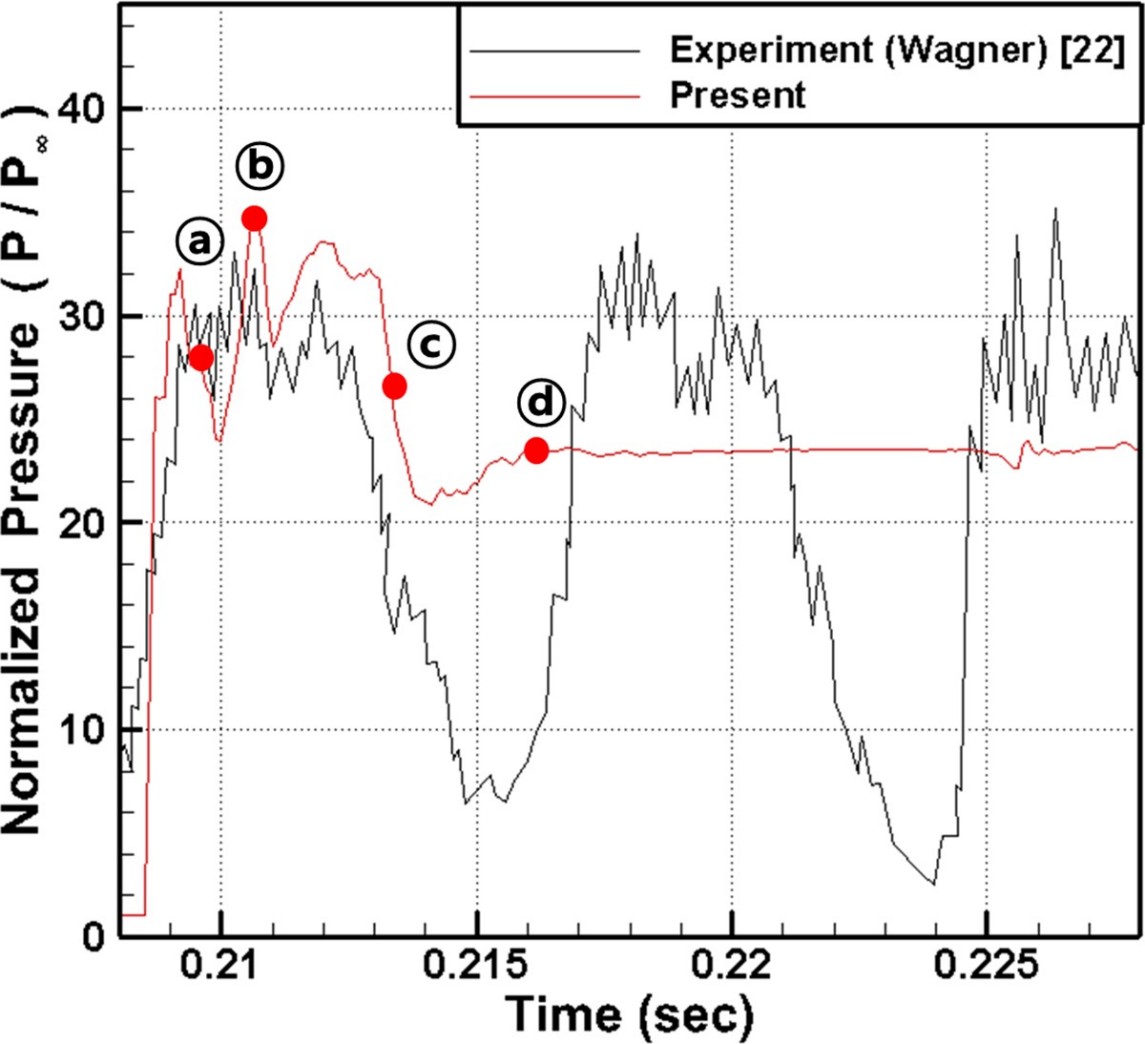
Comparison of pressure time history at x/h = 12.21 with experimental data.

Fig 8 shows the time history of the wall pressure near the flap of the isolator (x/h = 12.21) and a comparison with the experimental results for the high-amplitude oscillatory mode [22]. As shown in this figure, the pressure data from the experiments exhibit continuous fluctuations with time. In the present numerical results, the wall pressure initially increases and passes through point ⓐ, reaches the maximum at point ⓑ and decreases to point ⓒ. As shown in the figure, from point ⓐ to point ⓒ, the present results show good agreement with the experimental results. However, after approximately 0.214 sec, the pressure stops decreasing and starts to increase again. At pointⓓ (approximately at 0.216 sec), the pressure shows a constant value and remains at steady state, and these results are different from the experimental results.

**Fig 9:**
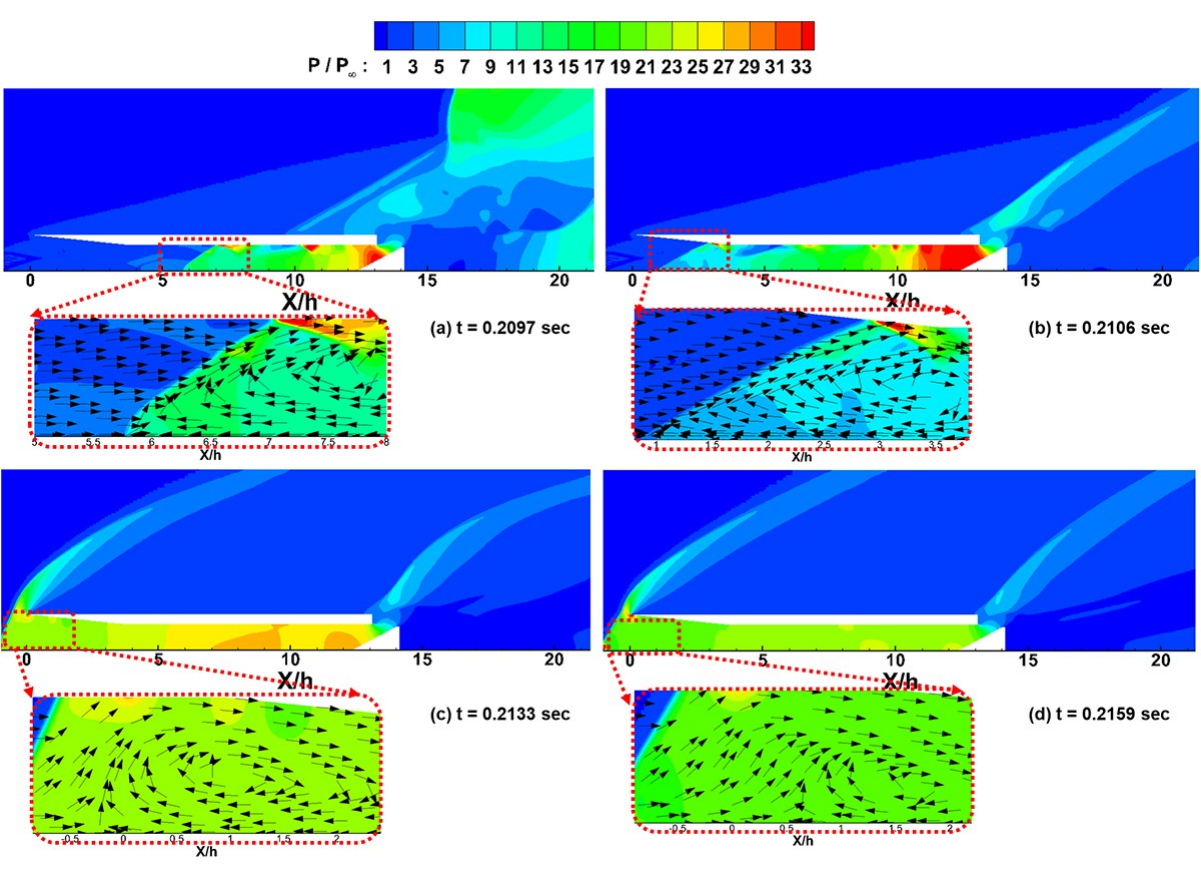
Normalized pressure contours for the inlet unstart case. (a) t = 0.2097 sec. (b) t = 0.2106 sec. (c) t = 0.2133 sec. (d) t = 0.2159 sec.

To study these differences in the results in detail, the normalized pressure distributions and velocity vectors in the inlet area are shown in Fig 9 at the moments of points ⓐ, ⓑ, ⓒ, and ⓓ in Fig 8.

As shown in Fig 9(A), due to the reduced flow path near the flap, the pressure is greater in this region. This greater pressure results in a shock wave that propagates upstream. As shown in the enlarged velocity vectors in Fig 9(A), due to the adverse pressure gradient, backflow occurs, collides with the incoming flow and results in a recirculation zone.

Fig 9(B) shows that the high-pressure zone near the flap is more widely distributed, and the shock wave propagates further upstream to the inlet cowl area. Additionally, it can be observed in the velocity vector distribution that the recirculation zone is still sustained.

Fig 9(C) shows that as the backflow reaches the front of the inlet, the high-pressure zone in the isolator is changed to a lower and wider zone. Furthermore, the shock wave reaches the inlet boundary of the computational domain, and the angle of the shock wave becomes greater than that shown in Fig 9(A) and Fig 9(B). As before, the velocity vector distribution shows that the recirculation zone is still sustained near the shock wave.

Fig 9(D) shows that, although the pressure in the isolator has been substantially reduced, the shock wave still stays at the inlet boundary of the computational domain. The velocity vector distribution shows that the recirculation zone is still sustained near the shock wave as well. This finding is because the backflow from the inside of the inlet is blocked by the incoming flow from the boundary of the computational domain due to the compulsory boundary condition. As observed in Fig 8, after the time of point ⓓ, the flow reaches a steady state and maintains the same configurations as those shown in Fig 9(D).

To summarize the results shown in Figs 8 and 9, the shock wave is generated near the flap due to the reduced flow path and propagates upstream. Until the shock wave reaches the boundary of the computational domain, the present results are in good agreement with the experimental results. However, after reaching the boundary, the shock wave cannot escape out of the inlet due to the compulsory inflow boundary condition, and thereby the flow leakage and the pressure reduction within the inlet are prevented.

### Flow analysis of inlet unstart (Domain 3)

In a hypersonic inlet, the processes of the buzz phenomenon can be categorized into the generation of the high-pressure zone in the isolator, the existence of backflow due to the adverse pressure gradient, the reduction in the isolator pressure and the inlet restart [8, 19–20]. In the previous section, it was shown that the main cause of the discrepancy between the present results and the experimental results was the prevention of flow leakage and pressure reduction within the inlet due to the limited computational domain. Therefore, in this section, to improve the accuracy of the present results, additional numerical analysis is performed using Domain 3 (x/h = -8.6~21.38), the extended computational domain that includes the region near the Mach nozzle exit. All the conditions are the same as those in the previous section except for the computational domain.

**Fig 10:**
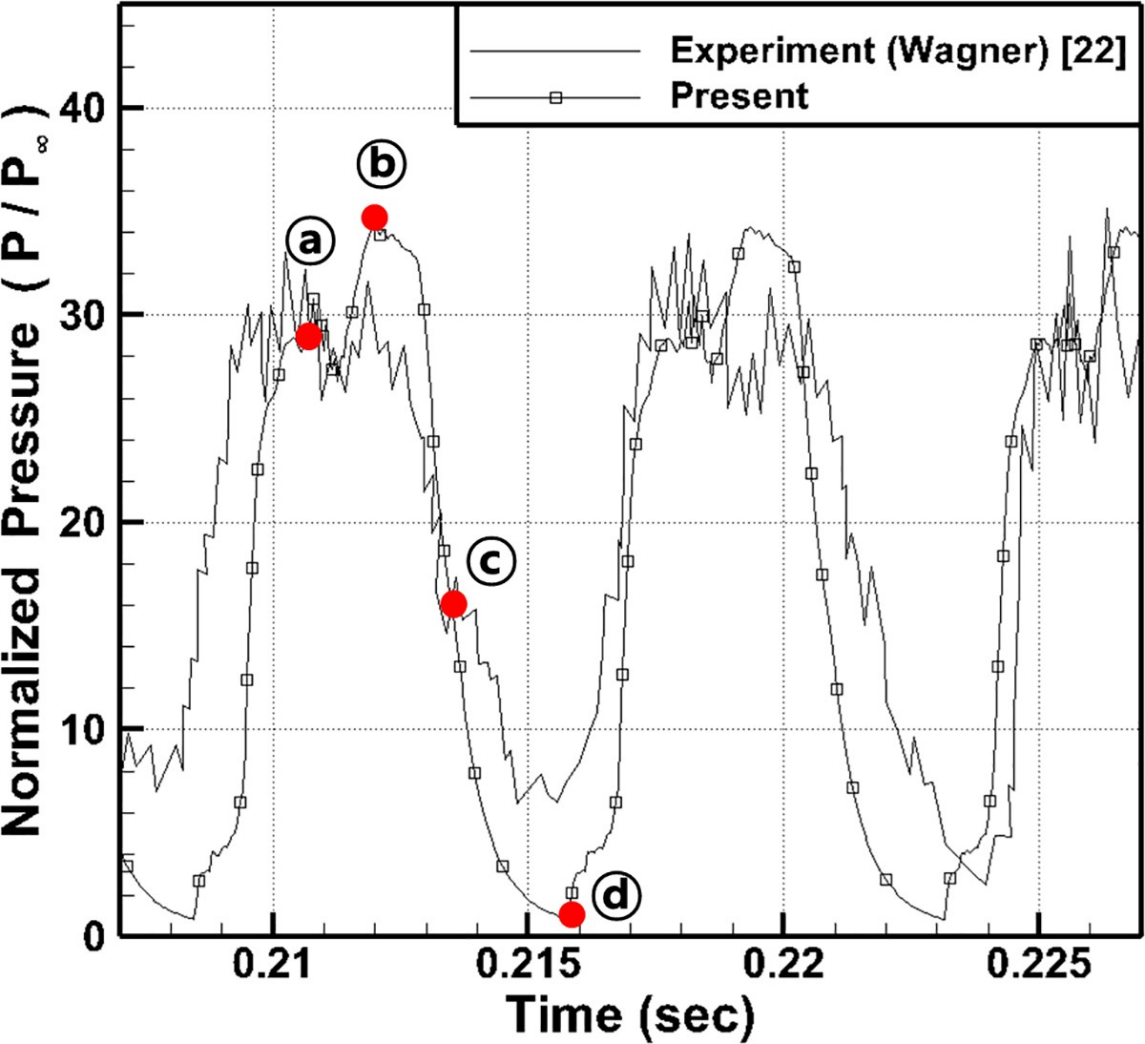
Comparison of pressure time history at x/h = 12.21 with experimental data.

Fig 10 shows the time history of the wall pressure near the flap of the isolator (x/h = 12.21) and a comparison with the experimental results. As shown in this figure, unlike in Fig 8, the pressure is completely reduced at point ⓓ, and the pressure oscillations occur in the same way as those in the experimental results.

Furthermore, the frequency and the amplitude of the pressure oscillations are very similar to those in the experimental results, and therefore, the present results are in good agreement with the experimental results.

**Fig 11:**
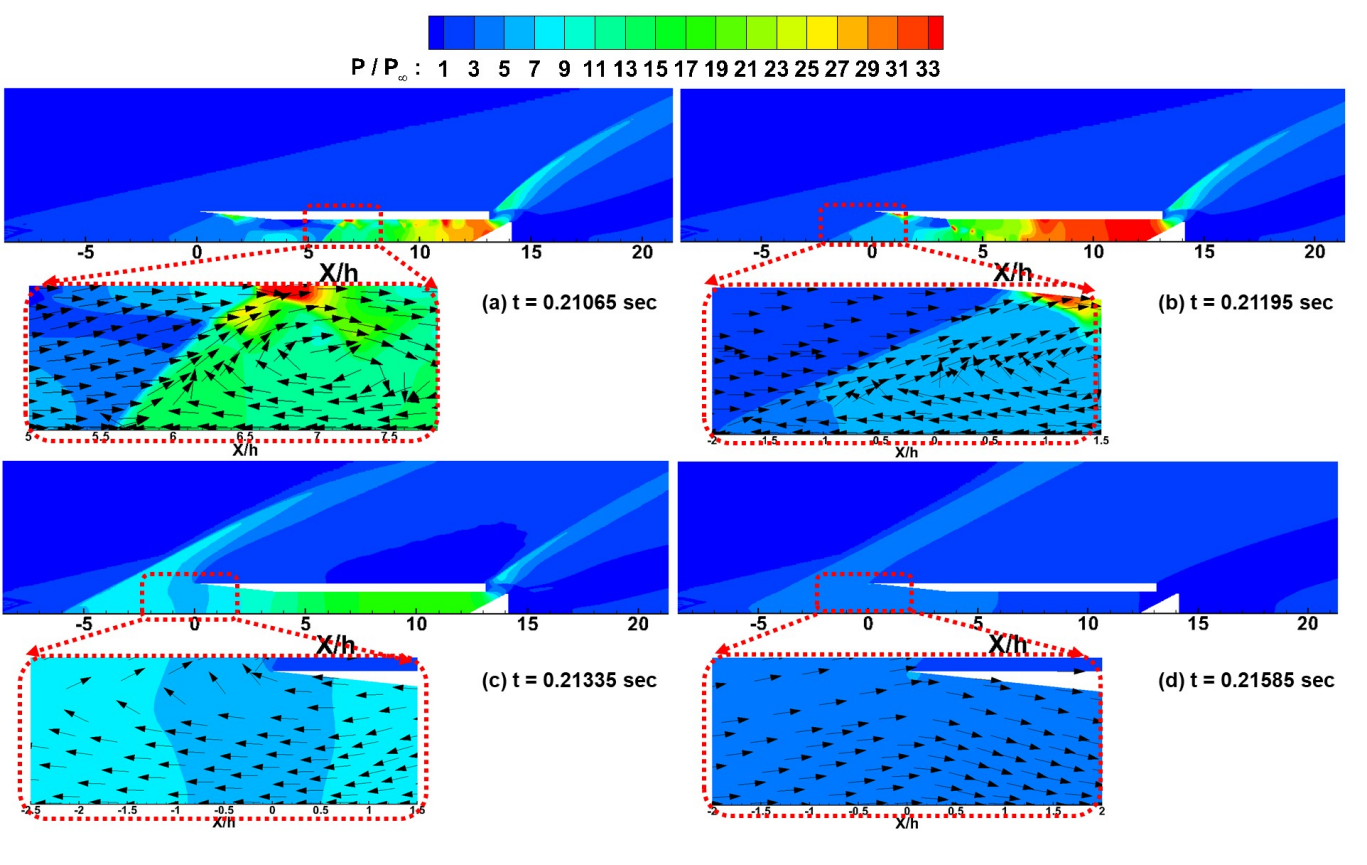
Normalized pressure contours for the inlet unstart case. (a) t = 0.21065 sec. (b) t = 0.21195 sec. (c) t = 0.21335 sec. (d) t = 0.21585 sec.

To study the flow characteristics with the pressure oscillations within the model in detail, the normalized pressure distributions and velocity vectors in the inlet area are shown in Fig 11 at the moments of points ⓐ, ⓑ, ⓒ, and ⓓ in Fig 10.

As shown in Fig 11(A) and 11(B), the pressure increase due to the reduced flow path by the flap and the shock wave propagation upstream are similar to the results shown in Fig 9(A) and 9(B). Additionally, in the enlarged velocity vectors, the recirculation zone is generated near the shock wave in the same way as shown in Fig 9(A) and 9(B).

However, after that, as shown in Fig 11(C), the high-pressure zone near the flap has been significantly reduced compared to that shown in Fig 9(C). Furthermore, the shock wave within the inlet propagates further upstream of the inlet. Therefore, the backflow is not interrupted by the inflow boundary condition, and the backflow escapes out of the inlet.

Fig 11(D) shows that the pressure within the inlet has almost been reduced to the free-stream pressure level, and the adverse pressure gradient has changed to a favorable pressure gradient. Additionally, as shown in the velocity vector distribution, the flow re-enters the inlet due to the newly obtained favorable pressure gradient.

After the time of point ⓓ, due to the re-entry of the flow, the high-pressure zone is regenerated near the flap, and the flow pattern returns to the result shown in Fig 11(A). In the subsequent results, the buzz processes, such as the generation of the high-pressure zone, the backflow due to the adverse pressure gradient, the isolator pressure reduction and the inlet restart, are repeated as shown in Fig 11(B)–11(D). These results can be regarded as typical inlet buzz patterns in hypersonic inlets [19, 20].

For the inlet start case described in section 3.1, the numerical results with a limited computational domain were in good agreement with the experimental results because the interactions between the upstream and inside of the inlet were not severe. However, for the cases of inlet unstart and the buzz phenomenon, the shock wave from the inside of the inlet propagates upstream, and backflow occurs; therefore, the interactions between the upstream and inside of the inlet can be intensive. Therefore, for the cases of inlet unstart and the buzz phenomenon, the computational domain must be wide enough to consider the active interactions between the upstream and inside of the inlet.

### Effects of boundary layer profiles

Due to their interactions with shock waves, boundary layer profiles can be an important factor in the inlet start and unstart phenomena. In the previous sections, a 19.05 mm-thick turbulent boundary layer was included in the inflow as was measured in the experiments [22]. To determine the effects of the boundary layer on inlet unstart phenomena, additional numerical analysis is performed with different boundary layer conditions using Domain 3.

**Fig 12:**
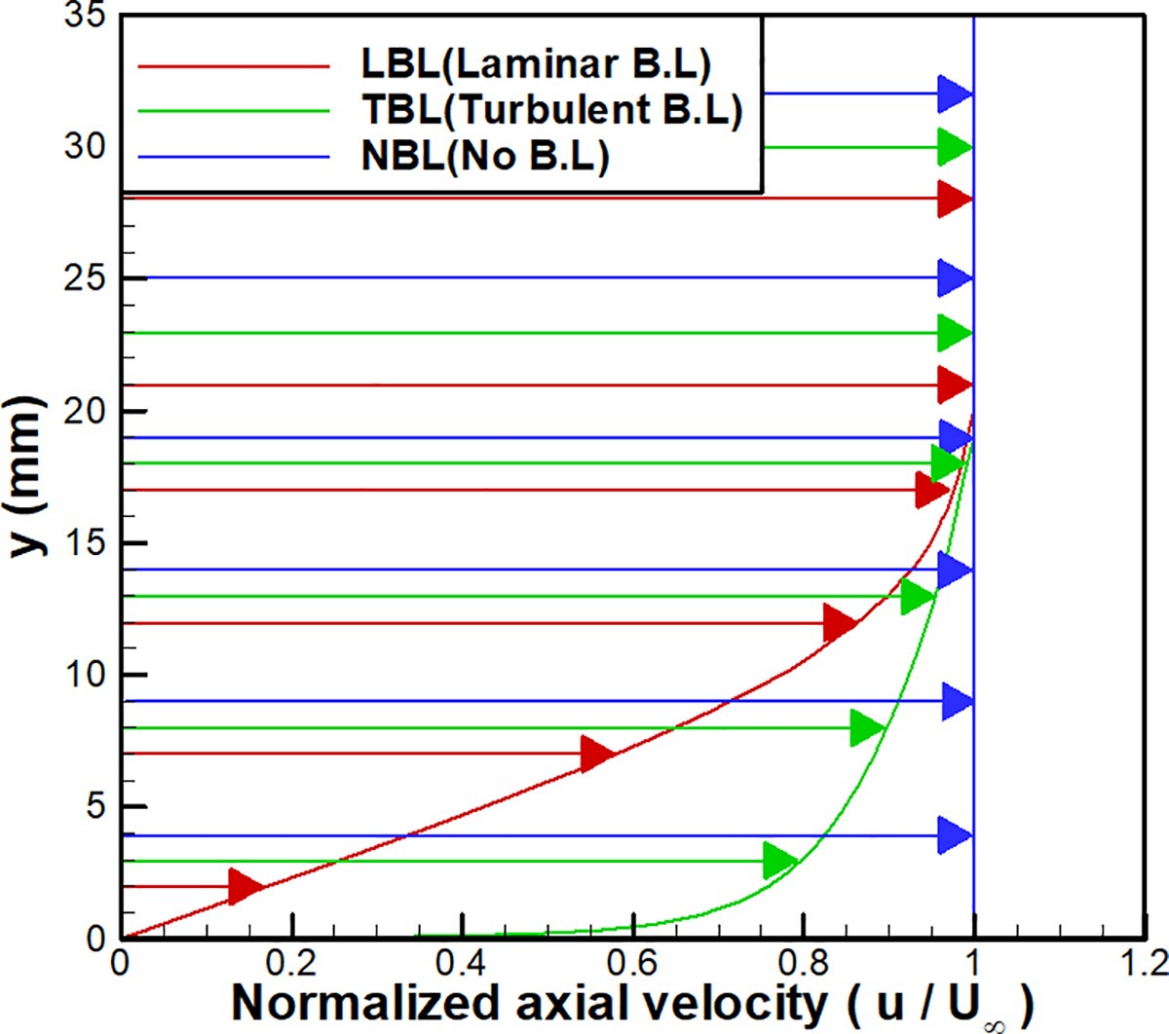
Velocity profiles for the laminar, turbulent and no boundary layer cases.

Fig 12 shows the three boundary layer profiles used in the present numerical analysis (laminar, turbulent and no boundary layer). In the figure, the thicknesses of the laminar and turbulent boundary layers are fixed at 19.05 mm. The boundary layer profiles are calculated by using classical boundary layer theory [31, 35]. As shown in the figure, the low-velocity region near the wall is thick for the laminar boundary layer case, whereas it is thin for the turbulent boundary layer case. There is no low-velocity region in the case without a boundary layer. For simplicity, the case with the laminar boundary layer is denoted as LBL, the case with the turbulent boundary layer is denoted as TBL, and the case with no boundary layer is denoted as NBL.

**Fig 13:**
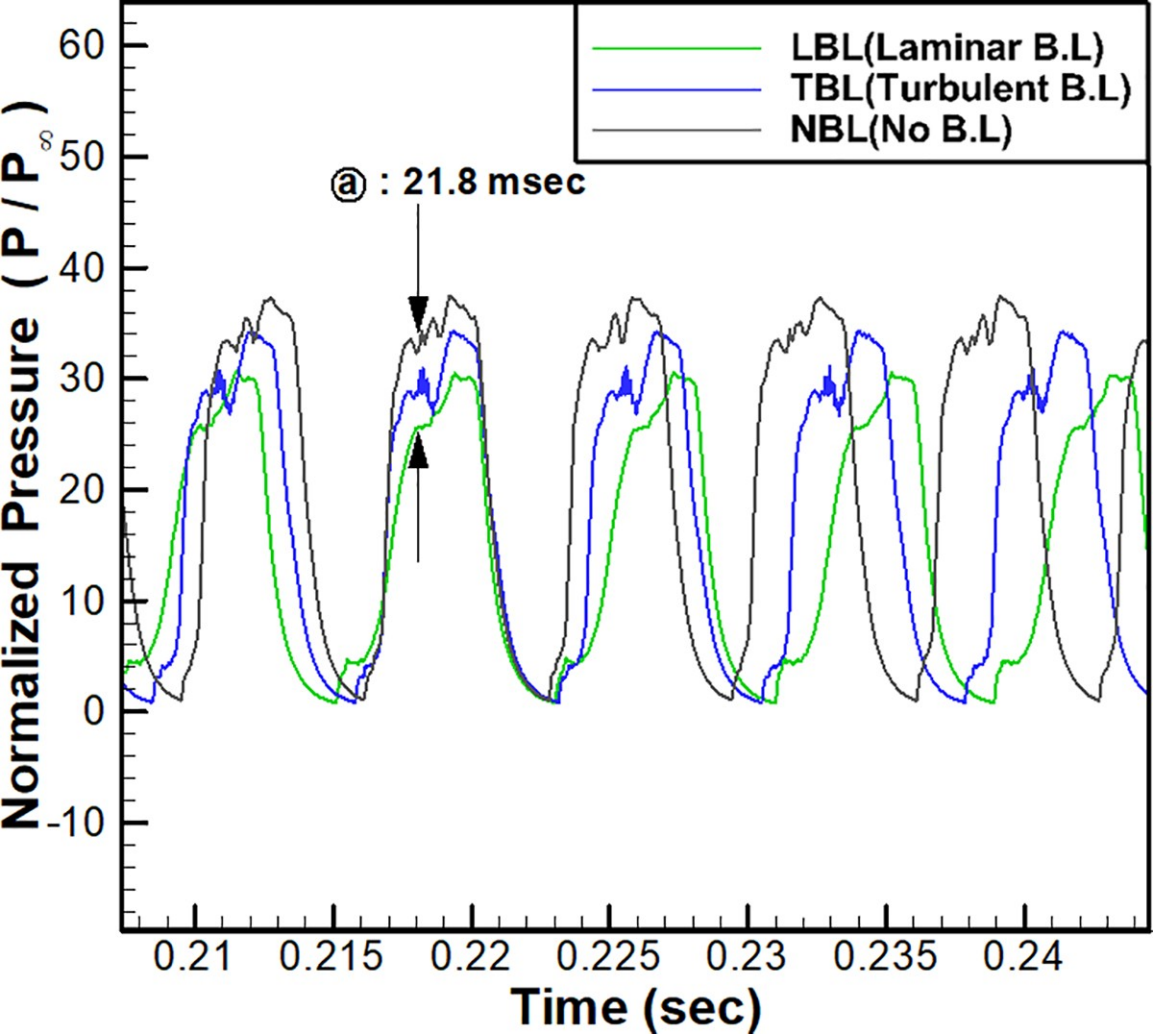
Comparison among the pressure time histories at x/h = 12.21 for the different boundary layer cases.

Fig 13 shows the time histories of the wall pressure near the flap (x/h = 12.21) for the cases with three different boundary layer conditions. As shown in the figure, three cases are in good agreement overall, but the period and amplitude of the oscillations slightly differ among the cases. For LBL, a smaller amplitude and longer period are shown, whereas a larger amplitude and shorter period are shown for NBL. In other words, a larger low-velocity region in the inflow results in a smaller oscillation amplitude and a longer oscillation period.

For detailed analysis, the frequencies and the typical maximum and minimum pressures of the oscillations in Fig 13 are summarized in Table 2. As shown in the table, the case with the largest low-velocity region, LBL, exhibits the lowest frequency (the longest period) among the three cases. Decreasing the low-velocity region as in TBL and NBL results in an increase in the frequency. Furthermore, LBL also shows the lowest typical maximum pressure among the three cases, and it increases as the low-velocity region decreases for TBL and NBL. However, the typical minimum pressures of the oscillations are similar at the free stream pressure for all cases, which is consistent with the moment of complete pressure relief illustrated in Fig 11(D).

**Table 2:** Oscillation characteristics for different boundary layer cases.

	Laminar boundary layer (LBL)	Turbulent boundary layer (TBL)	No boundary layer (NBL)
Frequency	127 Hz	135 Hz	151 Hz
Typical minimum pressure, P_min_/P_0_	0.9	0.9	1.1
Typical maximum pressure, P_max_/P_0_	30.4	34.1	37.2

**Fig 14:**
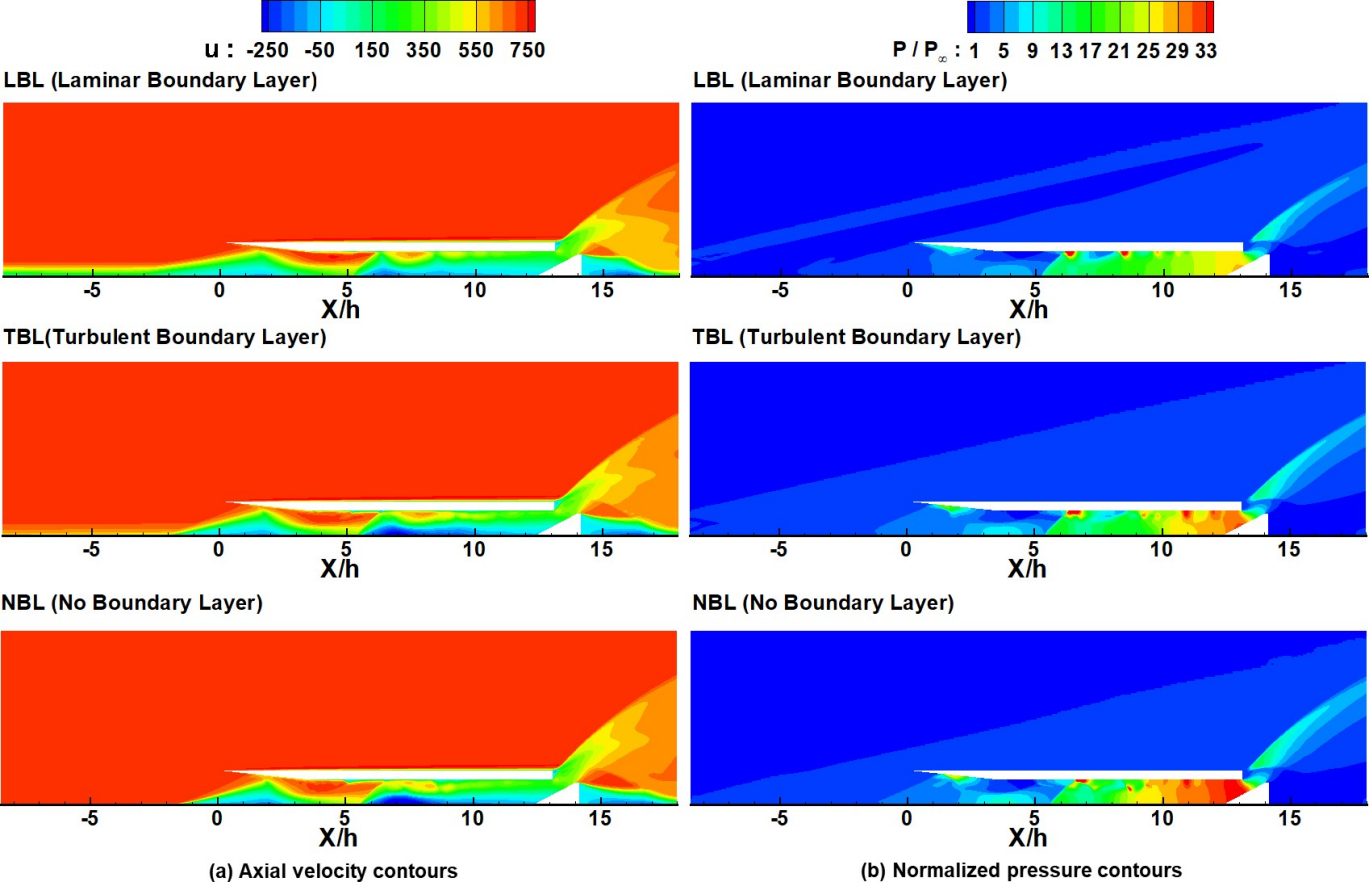
Velocity contours and normalized pressure contours at t = 21.8 msec for different boundary layer cases. (a) Axial velocity contours. (b) Normalized pressure contours.

For further detailed observation, the axial velocities and the normalized pressure distributions for the three cases are shown in Fig 14 at the moment of point ⓐ (t = 21. 8 msec) in Fig 13. As shown in Fig 14(A), LBL shows the largest low-velocity region at the inflow boundary near the bottom wall, which means the smallest amount of air enters the inlet. However, in the figure, there is no low-velocity region at the inflow boundary for NBL, so the largest amount of air enters the inlet for NBL among the three cases. As shown in Eq 1, the total pressure of the choked mass flow near the flap is proportional to the amount of the inflow.

pt=m˙A*RTtγ(γ+12)(γ+1)(2−2γ) (1)

Therefore, as shown in Fig 14(B), LBL shows the lowest pressure near the flap, whereas NBL shows the highest among the three cases. Furthermore, as indicated in Fig 14(A), because of the high pressure near the flap, the backflow observed for both TBL and NBL is remarkable. Therefore, due to the increased backflow for TBL and NBL, the period of the oscillation decreases and the frequency increases, as shown in Table 2.

These characteristics of the pressure oscillations are consistent with the experimental results of Tan et al [7]. In their results, the frequency and the typical maximum of the pressure oscillations increased with the throttling ratio. As shown in Eq 1, the effects of an increase in the throttling ratio (a decrease in the choked area) on the total pressure are similar to those of an increase in the inflow mass flow rate. Therefore, these characteristics of the frequencies and amplitudes of the oscillations can be explained by the same principles.

Therefore, for the investigation of inlet unstart phenomena, an estimation of the boundary layer in the inflow should be accurate to predict the pressure oscillations correctly.

## Conclusion

In the present study, the flow characteristics of the inlet start and unstart phenomena in a scramjet inlet-isolator test model were investigated using numerical analysis. The RANS-based OpenFOAM solver was used for numerical analysis, and the k-ω SST model was applied to consider the turbulent effect.

In the results, for the case of inlet start without the flap at the end of the model, the results of the present study were in good agreement with the existing experimental results and the LES results. The results for inlet unstart case with an extended computational domain were also in good agreement with the experimental results. The typical buzz phenomena, such as high-pressure generation, backflow, pressure reduction and inlet restart, were accurately reproduced.

For the cases with different boundary layer profiles, the large low-velocity region at the inflow boundary resulted in a small amount of air inflow and a decrease in the frequency and amplitude of pressure oscillations. To acquire accurate predictions of the frequencies and amplitudes of pressure oscillations, accurate boundary layer profiles should be provided.
